# Real World Outcomes of Pembrolizumab and Reduced-Dose Lenvatinib in Recurrent Endometrial Cancer by Platinum and p53 Status

**DOI:** 10.1016/j.gore.2026.102084

**Published:** 2026-04-15

**Authors:** Alexa Dzienny, Yasmin Abozenah, James Griffith, Sidra Sohail, Yifang Eva Pan, Christina Vlamis, Katelyn Yamartino, Jessica Leclair, Gary Israel, Michelle Greenman, Blair McNamara, Masoud Azodi, Elena S. Ratner, Alessandro Santin, Peter Dottino, Katyayani Papatla, Aparna Kailasam, Pei Hui, Natalia Buza, Gary Altwerger

**Affiliations:** a Yale University School of Medicine, Department of Obstetrics, Gynecology and Reproductive Science, New Haven, CT 06520, United States; b University of Chicago, Pritzker School of Medicine, Department of Obstetrics and Gynecology, United States; c University of Connecticut School of Medicine, Department of Obstetrics and Gynecology, Farmington, CT 06030, United States; d Yale University School of Medicine, Department of Department of Radiology and Biomedical Imaging, New Haven, CT 06520, United States; e Yale University School of Medicine, Department of Pathology, New Haven, CT 06520, United States; f University of Chicago, Department of Obstetrics and Gynecology, Chicago, IL 60637, United States

**Keywords:** Pembrolizumab, Lenvatinib, Endometrial cancer, Uterine carcinosarcoma

## Abstract

**Objectives:**

The objective of this study was to evaluate the efficacy of pembrolizumab and lenvatinib in recurrent endometrial cancer (EC) by platinum resistance status and p53 status, and to compare outcomes between patients who started lenvatinib at doses of 10 mg or less versus standard starting doses.

**Methods:**

A retrospective study was conducted in patients with recurrent EC treated with pembrolizumab and lenvatinib between March 2019 and February 2023. Primary outcomes were objective response rate (ORR) and progression-free survival (PFS) by platinum status, p53 status, and lenvatinib dose. Secondary outcomes included adverse events and lenvatinib-related toxicity. Noninferiority was assessed using the Wald confidence interval and a propensity score–matched cohort.

**Results:**

Among 59 patients, 85% had high-grade histology, 75% were platinum-resistant, and 67.8% harbored p53 mutations. The overall ORR was 37%. There were no significant differences in ORR or PFS by grade, platinum status, or starting dose. On multivariable analysis, p53 mutation and ECOG < 2 were independently associated with improved PFS. The 10 mg lenvatinib dose demonstrated the longest median treatment duration (74 days) with improved tolerability. When stratifing lenvatinib starting dose as ≤ 10 mg versus > 10 mg, ORR was 50% and 26%, respectively. The lower dose group met criteria for noninferiority.

**Conclusions:**

In a heavily pretreated, high grade, platinum resistant population, pembrolizumab and lenvatinib showed activity comparable to prior studies despite a more resistant cohort. Reduced lenvatinib doses of 10 mg or less were associated with noninferior ORR and lower toxicity, supporting dose optimization. In addition, p53 mutation was independently associated with prolonged PFS, suggesting it may be a biomarker of response.

**KEY MESSAGES:**

What is already known on this topic:

Pembrolizumab plus lenvatinib has been an emerging treatment for recurrent endometrial cancer, showing survival benefit in pMMR tumors. However, most data are from clinical trials with less heavily pretreated patients, and uncertainty remains about efficacy in platinum-resistant, p53-mutated, high-grade tumors and the optimal starting dose of lenvatinib given its toxicity.

What this study adds:

In a real-world cohort enriched for platinum-resistant, high-grade, p53-mutated, heavily pre-treated endometrial cancers, lenvatinib plus pembrolizumab achieved an objective response rate consistent with prior trials. A reduced starting dose of lenvatinib (≤10 mg) was non-inferior to higher doses, better tolerated, and associated with fewer dose reductions. Additionally, p53 mutation and good ECOG status predicted improved progression-free survival.

How this study might affect research, practice or policy:

These findings suggest that reduced-dose lenvatinib may maintain efficacy while improving tolerability in heavily pretreated high-grade tumors and platinum resistant, p53 mutated patients, supporting individualized dosing strategies. They also highlight p53 mutation as a potential predictive biomarker, warranting larger studies.

## 1 Introduction

Endometrial cancer is the most common gynecologic malignancy in the United States, and its incidence continues to rise, with a reported annual incidence of 28.3 new cases per 100,000 between 2018 and 2022 (Cancer of the Endometrium - Cancer Stat Facts. SEER. Accessed June 22, 2025). Recurrences are associated with poor outcomes, with a median survival of approximately 14 months. Effective treatment options for aggressive histology, advanced-stage disease, or recurrence, remain limited, highlighting the need for effective treatment options.

Immunotherapy has recently emerged as a promising treatment option for advanced or recurrent endometrial cancer. Pembrolizumab, an immune checkpoint inhibitor, has demonstrated activity in advanced and recurrent endometrial cancer. This is particularly effective in solid tumors with mismatch repair deficiency. With these findings, the FDA approved pembrolizumab for endometrial cancer patients with mismatch repair-deficient (dMMR) (Ott et al., 2017, Marabelle et al., 2020).

However, single-agent immunotherapy provides benefit in only 20–30% of uterine tumors, specifically those with MSI/dMMR (Makker et al., 2020, Makker et al., 2023). To expand treatment options, a phase III trial (Keynote 775/ study 309) addressed the pMMR population by comparing lenvatinib plus pembrolizumab to non-platinum-based chemotherapy regimen in patients with advanced endometrial cancer who received one prior platinum-based chemotherapy. The trial demonstrated that combining an immune checkpoint inhibitor with lenvatinib, a targeted tyrosine kinase inhibitor, enhanced antitumor activity regardless of the MSI/dMMR status. In pMMR tumors, this combination significantly improved progression-free survival and overall survival (OS) compared to non-platinum chemotherapy (Makker et al., 2023, Makker et al., 2022). The National Comprehensive Cancer Network (NCCN) guidelines now recommend lenvatinib, combined with pembrolizumab for pMMR tumors (NCCN Guidelines for Patients, 2023).

While combination lenvatinib-pembrolizumab regimen has expanded treatment options, some limitations remain. These include high toxicity rates associated with lenvatinib, limited data on survival outcomes, and uncertainty about the regimen’s efficacy in heavily pretreated, platinum-resistant tumors with high-grade, p53 mutant histology and carcinosarcoma. Although the recommended starting dose of lenvatinib is 20 mg, many patients experience dose interruptions, reductions, or treatment discontinuation due to adverse effects. A retrospective study by How et al. found that starting lenvatinib at 14 mg was associated with fewer dose reductions due to toxicity and a longer median time to treatment interruption. Importantly, no significant differences in objective response rate, progression-free survival, or overall survival (OS) were observed between patients who started at 20 mg or 14 mg (How et al., 2021). Notably, How et al. did not specify whether patients were platinum-resistant or provide information on p53 mutation status, which limits the generalizability of their findings to endometrial tumors with known resistance profiles.

The KEYNOTE-775 trial included patients with recurrence or progression after one prior platinum-based regimen but did not stratify outcomes by platinum sensitivity or resistance. To further assess the role of pembrolizumab and lenvatinib in platinum sensitive disease, Wang et al. conducted a retrospective study comparing survival between carboplatin paclitaxel rechallenge and pembrolizumab plus lenvatinib in patients with advanced or recurrent endometrial cancer whose prior platinum therapy had been completed at least 6 months earlier. Survival did not differ between the groups, suggesting that either platinum rechallenge or pembrolizumab plus lenvatinib may be reasonable second line options in platinum sensitive recurrence (Wang et al., 2025, Marth et al., 2025).

Given pembrolizumab and lenvatinib's toxicities, questions remain about the regimen’s viability in heavily pretreated, platinum-resistant, P53 mutant patients, and whether they would benefit from reduced dose pembrolizumab-lenvatinib. This retrospective review aims to investigate the clinical efficacy of pembrolizumab and lenvatinib combination therapy in patients with high grade, P53 mutated, heavily pretreated, platinum-resistant tumors at a reduced dose. At our institution, lenvatinib is often initiated at a reduced starting dose of 10 mg due to concerns about toxicity and tolerability in heavily pretreated patients. In this context, we aimed to evaluate the efficacy of lenvatinib in combination with pembrolizumab when administered at a starting dose of ≤ 10 mg. This retrospective analysis provides real-world evidence of pembrolizumab–lenvatinib efficacy in heavily pretreated, platinum-resistant, p53-mutated endometrial cancers at a reduced dose.

## 2 Methods

### 2.1 Patient population

We conducted a retrospective cohort study of patients with recurrent endometrial cancer treated with pembrolizumab and lenvatinib at a single academic tertiary center from March 2019 to February 2023. Inclusion criteria were platinum-resistant and platinum-sensitive patients, resistance was defined as recurrence or progression within 6 months of completing prior platinum-based therapy. Platinum-sensitive disease was defined as recurrence or progression 6 months or more after completion of prior platinum-based therapy. Patients were excluded if they had incomplete medical records at our institution. Treatment recommendations and lenvatinib starting dose were made at the discretion of attending gynecologic oncologists based on clinical judgment, number of prior lines of therapy, ECOG, prior treatment tolerance, and anticipated ability to tolerate therapy, particularly given the known toxicity profile of lenvatinib. Institutional Review Board (Protocol 2000028584) approval was obtained and written informed consent was exempt by the Yale Human Investigations Committee.

### 2.2 Clinical data collection

Electronic medical records were reviewed for baseline demographic data, including age, race and ethnicity, ECOG status, number of prior lines of therapy, stage at diagnosis, tumor histology, tumor mutational burden, microsatellite instability status, P53 and platinum status.

The primary objective was to evaluate the clinical response to pembrolizumab and lenvatinib based on tumor grade, platinum status, and starting lenvatinib dose using the following endpoints: objective response rate and progression-free survival. High-grade tumors were defined as high-grade endometrioid, serous, clear cell, carcinosarcoma, and mixed histologic subtypes. P53 status was defined by the institutional pathology criteria.

Objective response rate was evaluated through computed tomography of the chest, abdomen, and pelvis, performed every 3 months during treatment. Radiological images were independently reviewed by a gynecologic oncologist and a radiologist. Treatment responses were categorized as complete response (CR), partial response (PR), stable disease (SD), or progressive disease (PD). Patients achieving a complete or partial response were classified as responders. Patients with stable disease, progressive disease, or who discontinued treatment prior to achieving an objective response were classified as non-responders. Progression-free survival was defined as the time from initiation of pembrolizumab and lenvatinib to radiographic progression or death, whichever occurred first.

Data was collected including duration of treatment with pembrolizumab and lenvatinib, interruptions of treatment, starting lenvatinib dose, dose reductions, reasons for discontinuation, hospitalizations, immunotoxicity, adverse events, response to treatment, and death.

### 2.3 Statistical analysis

Survival outcomes were evaluated using Kaplan–Meier survival curves, and statistical analysis was conducted using GraphPad Prism v10.4.0 (527). Statistical significance was defined as a p-value < 0.05. Exploratory multivariable Cox regression analysis was performed to assess associations between selected clinical variables and progression-free survival, recognizing limitations related to sample size. In this model, lenvatinib starting dose was treated as a continuous variable, whereas dose-stratified analyses evaluated lenvatinib starting dose as a binary variable (≤10 mg vs > 10 mg). Fisher’s Exact test was used to compare objective response rates between independent groups.

Secondary outcomes included lenvatinib tolerance and related toxicities. Clinical efficacy of initiating a lower lenvatinib dose was further evaluated using non-inferiority analysis comparing the objective response rate between patients who started on ≤ 10 mg (reduced dose group) versus > 10 mg of lenvatinib. An exploratory non-inferiority framework was applied for hypothesis generation, recognizing the limitations of the retrospective design and sample size. The absolute difference in objective response rate between groups and its corresponding 95% confidence interval were calculated. A strict prespecified non inferiority margin of 2% was selected, as a difference of less than 2% (in the direction of > 10 mg group) between the reduced dose group and the > 10 mg dose group was considered not clinically meaningful. The Wald confidence interval for the difference in objective response rate was then used to assess non inferiority using a one tailed alpha level (van den Heerik et al., 2026). As a sensitivity analysis, we also used the *nearest neighbor* method to match cases in the ≤ 10 mg versus > 10 mg of Lenvatinib groups using the MatchIt (Ho et al., 2011) package of R, using the following variables: dMMR status, tumor grade, platinum status, ECOG scores, age and BMI. The matched dataset was then used to calculate and evaluate the risk ratio in the matched sample using a generalized linear model with a quasibinomial response (response vs. non-response).

## 3 Results

Patient baseline characteristics and clinicopathologic information were collected (Table 1). Baseline clinical characteristics stratified by lenvatinib starting dose (≤10 mg vs > 10 mg), including ECOG performance status, are shown in Supplementary Table S1. The median age of the study population was 68 years old. Most patients had an ECOG performance status of 0 or 1 (86%, n = 51), and 14% had an ECOG status of 2. Histology included serous (39%), endometrioid (25%), clear cell (5%), carcinosarcoma (19%), P53 mutated (67.8%) and mixed histologies (10%) (Table 1). High grade histologies were present in 46 patients (77.9%), whereas 13 patients (22.1%) had low-grade endometrioid histology. Eighty-eight percent (n = 52) were mismatch repair proficient (pMMR), and 10% (n = 6) were mismatch repair deficient (dMMR) and 1 patient (2%) had unknown MMR status. Fifteen patients (25%) were platinum-sensitive, and 44 (75%) were platinum-resistant. Forty-one patients (69%) had received two or more prior lines of therapy, with a median number of prior treatments of 2 (range: 1–11). Thirty-one patients (52.5%) started lenvatinib at > 10 mg, while 28 patients (47%) received a starting dose of ≤ 10 mg.

**Table 1:** Baseline Characteristics and Clinicopathologic data, n = 59.

Age (years), median range	68 (51–87)
BMI, median (IQR)	27.3 (15.8–68.2)
ECOG PS, n (%)	
0	28 (47%)
1	23 (39%)
2	8 (14%)
3–5	0
Stage at Diagnosis, n (%)	
I	14 (24%)
II	3 (5%)
III	17 (29%)
IV	24 (41%)
Histology, n(%)	
Serous	23 (39%)
Endometrioid	15 (25%)
Clear cell	3 (5%)
Carcinosarcoma	11 (19%)
Mixed	6 (10%)
Other (cervical, ovarian, stromal carcinoma)	1 (2%)
Grade	
Low Grade	13 (22.1%)
High Grade	46 (77.9%)
Mismatch Repair Status	
Mismatch Repair Proficient	52 (88%)
Mismatch Repair Deficient	6 (10%)
*Unknown	1 (2%)
Platinum Status, n(%)	
Sensitive	15 (25%)
Resistant	44 (75%)
Prior Lines of Therapy, n (%)	
1 prior line of therapy	18 (31%)
2 prior lines of therapy	22 (37%)
3 or more prior lines of therapy	19 (32%)
p53 Status	
Wildtype	19 (32%)
Mutation	40 (67.8%)

**Fig. 1:**
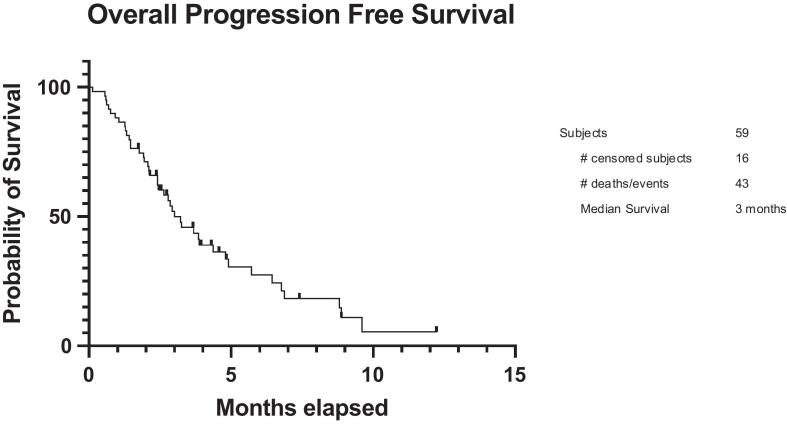
Overall PFS.

Among 59 patients, the objective response rate was 37%, with 18 partial responses and 4 complete responses, P53 mutants had an ORR of 42.5% and platinum resistant patients had an ORR of 34% (Table 2). No significant difference in objective response rate was seen based on platinum status, p53 status or ECOG status (Table 2). Patients starting with a Lenvatinib dose ≤ 10 mg had almost twice as favorable an objective response rate at 50%, compared to > 10 mg with an objective response rate of 25.8%, although this difference was not statistically significant (Table 2). The overall median progression-free survival was 3.0 months (Fig. 1). Among responders, the duration of response varied widely, with several patients demonstrating prolonged disease control beyond six months, and the longest observed response exceeding twelve months.

**Table 2:** Objective Response Rate of Endometrial Cancer with Pembrolizumab and Lenvatinib.

	n	Partial Response	Complete Response	Stable Disease	Progressive Disease	ORR
Overall	59	18	4	6	31	37%
Platinum Status						
Platinum Sensitive	15	7	0	0	8	47%
Platinum Resistant	44	11	4	6	23	34%
Lenvatinib Starting Dose						
≤ 10 mg	28	12	2	0	14	50%
>10 mg	31	6	2	6	17	25.8%
P53 Status						
P53 wildtype	19	5	0	1	13	26%
P53 mutant	40	13	4	5	18	42.5%
ECOG Status						
ECOG ≤ 1	51	17	4	6	24	41.2%
ECOG 2+	8	1	0	0	7	12.5%

ECOG: Eastern Cooperative Oncology Group.

The objective response rate varied by lenvatinib starting dose (Supplementary Data, Table S2), with highest objective response rate observed in those started at 10 mg (objective rate response = 52.4%, 9 partial responses and 2 complete responses). This was closely followed by the 8 mg subgroup, demonstrating an objective response rate of 50%. In contrast, patients starting at 12 mg (n = 6), 14 mg (n = 7) and 20 mg (n = 18) had a lower objective rate response of 33.3%, 28.6% and 22.2% respectively. The single patient treated at 4 mg did not respond, resulting in a 0% objective rate response. These findings suggest that starting doses in the range of 8–14 mg may achieve similar response rates compared to the recommended dose (20 mg).

To further explore the clinical efficacy of initiating a lower lenvatinib dose, we performed a non-inferiority test by dichotomizing the objective response rate between ≤ 10 mg versus > 10 mg lenvatinib subgroups. A strict non-inferiority margin of 2% was selected. With the objective response rate being 50.0% in the ≤ 10 mg group and 25.8% in the > 10 mg group, the absolute difference was found to be + 24.2% (95% CI: +0.040 to + 0.444, using a one-tailed alpha level) in favor of the lower dose (≤10 mg, Supplementary Data, Figure S3). Based on the confidence interval, one can reject the null hypothesis of non-inferiority. In addition, with the confidence interval lying above zero, these findings suggest that patients selected to receive reduced starting doses experienced more favorable outcomes relative to outcomes observed in patients receiving higher starting doses. We repeated this comparison on a matched dataset based on propensity scores (i.e., the propensity of being in the ≤ 10 mg group vs. the > 10 mg using the nearest neighbor method) (Ho et al., 2011). In this matched analysis, the difference (low dose – high dose) was 20.6% (95% CI: −0.015 to + 0.427, using a one-tailed alpha level); this interval did contain zero but did not contain −0.02 (the non-inferiority margin of 2% in favor of the high dose), reemphasizing that the reduced dose (≤10 mg) group was non-inferior to the > 10 mg dose group.

When stratified by starting lenvatinib dose, patients receiving ≤ 10 mg had a non-statistically significant progression-free survival compared with those receiving > 10 mg, with median progression-free survival of 3.22 months versus 2.93, respectively (HR: 0.790, 95% CI: 0.435–1.44) (Fig. 2a). No statistically significant differences in progression-free survival were observed when comparing additional dose cutoffs, including ≤ 14 mg versus > 14 mg and ≤ 20 mg versus > 20 mg (Supplementary Data, Figure S4a-S4b).

**Fig. 2a:**
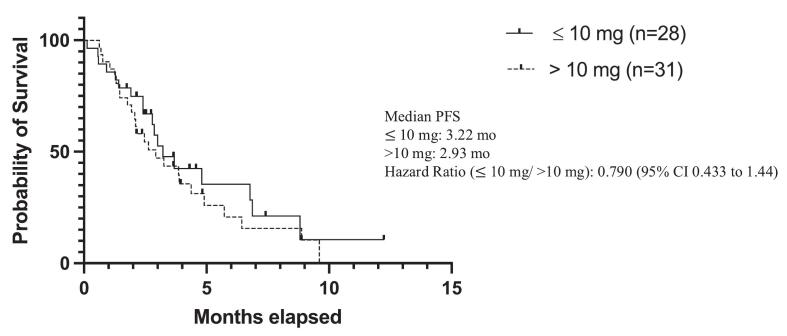
PFS Lenvatinib Starting Dose (≤10 mg vs. > 10 mg).

Eighty eight percent of patients were mismatch repair proficient (n = 52). There was no significant difference in progression-free survival between MMR-proficient and MMR-deficient patients (HR 0.540; 95% CI of 0.171 to 1.71) (Fig. 2b). There was no significant difference in progression-free survival based on platinum status either (HR 1.44, 95% CI 0.747 to 2.79) (Fig. 2c).

**Fig. 2b:**
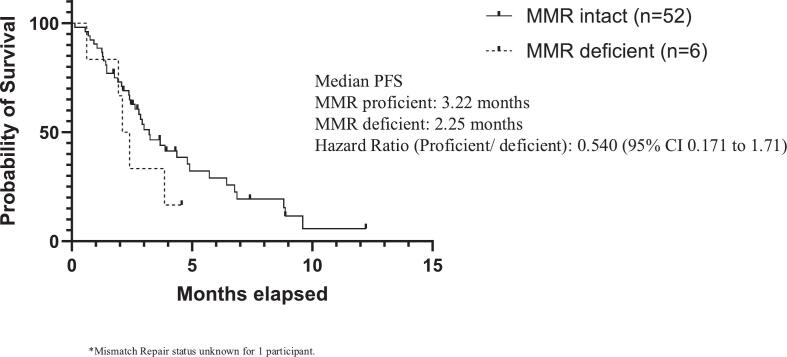
PFS Mismatch Repair Status.

**Fig. 2c:**
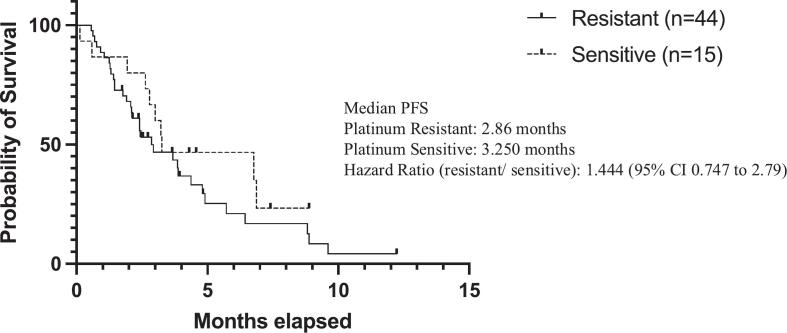
PFS Platinum Status.

In contrast, ECOG status and p53 mutation status were significantly associated with progression-free survival. Patients with ECOG scores < 2 had a significantly longer progression-free survival compared to those with ECOG ≥ 2 (HR: 0.145, 95% CI: 0.0431–0.487) (Fig. 2d). Patients harboring a p53 mutation had improved progression-free survival compared to those with wild-type p53 (HR: 0.356, 95% CI: 0.177–0.796) (Fig. 2e).

**Fig. 2d:**
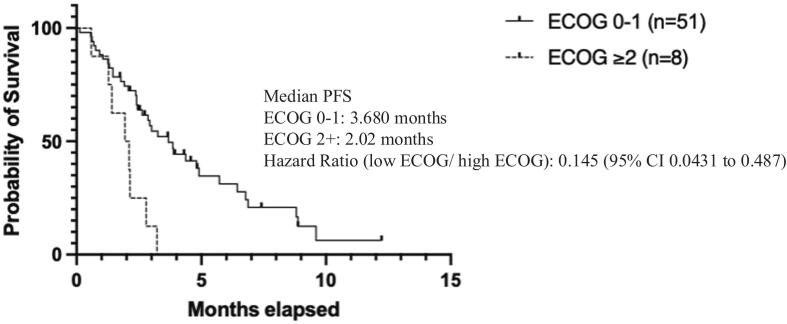
PFS ECOG Status.

**Fig. 2e:**
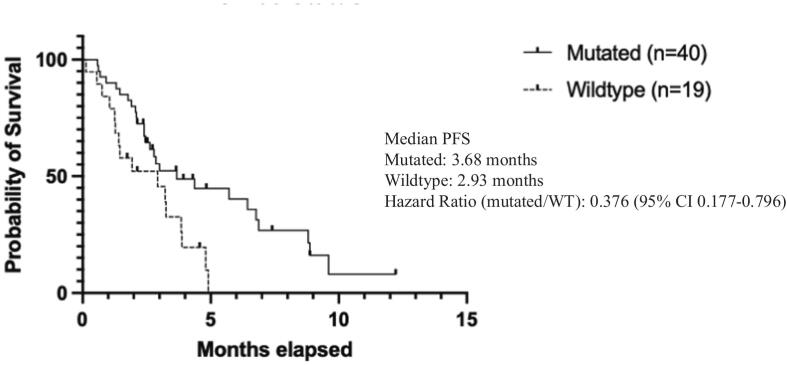
PFS p53 status.

In an exploratory multivariate Cox proportional hazard model, p53 mutation (HR: 0.459, 95% CI: 0.219–0.985, p = 0.0407) and ECOG < 2 (HR: 0.298, 95% CI: 0.115–0.814, p = 0.014) remained independently associated with improved progression-free survival. There were no statistically significant associations between progression-free survival and lenvatinib dose, number of prior lines of therapy, platinum resistance, age, or BMI in the model (Supplementary Data, Table S5).

Among the 59 patients included, lenvatinib-related adverse events and reasons for treatment discontinuation were evaluated and stratified by lenvatinib starting dose (>10 mg vs ≤ 10 mg) (Supplementary Data, Table S6). The most frequently reported adverse events overall were hypertension (42%), fatigue (41%), and diarrhea (25.4%), followed by anorexia (25.4%), nausea (20%), and musculoskeletal symptoms (17%). When stratified by starting dose, hypertension (52% vs 32%) and fatigue (45% vs 36%) were more common among patients receiving > 10 mg compared with those receiving ≤ 10 mg, whereas diarrhea was more frequently observed in the ≤ 10 mg group (39% vs 13%).

Less common adverse events included rash (8%), mucositis (3%), hand–foot syndrome (3%), and fistula (3%). Other toxicities observed in ≤ 3% of patients included headache, proteinuria, hives, voice changes/hoarseness, and elevated serum creatinine; all of these occurred exclusively in patients receiving > 10 mg as the starting dose (Supplementary Data, Table S6).

The most common reason for treatment discontinuation was disease progression, occurring in 40.7% of patients (n = 24) with more disease progression occurring in > 10 mg of lenvatinib. Discontinuation due to lenvatinib-related toxicity occurred in 18.6% (n = 11) with more than twice the number of patients discontinuing lenvatinib in the > 10 mg group (25.8%) when compared to the ≤ 10 mg lenvatinib group (10.7%), this was not statistically significant. Pembrolizumab-related toxicity overall caused 13.6% (n = 8) of treatment discontinuation, with a higher percentage of patients discontinuing treatment due to pembrolizumab-related toxicities in the less than or equal 10 mg lenvatinib group. Additional reasons for discontinuation included death (15.3%, n = 9) and patient relocation (1.7%, n = 1). At the time of data cutoff, 10.2% of patients (n = 6) remained on therapy (Supplementary Data, Table S6).

## 4 Discussion

### 4.1 Summary of main results

In our study, the overall response rate to lenvatinib plus pembrolizumab was 37%, consistent with previously reported data. Although the median progression-free survival was limited, this measure does not fully reflect the breadth of benefit observed, as a subset of patients experienced prolonged and durable responses to therapy. Notably, when compared to prior literature, our cohort included a higher proportion of patients with aggressive histologies and platinum resistance, interestingly there were also many patients with carcinosarcoma (19%, n = 11). Importantly, we found there was no significant difference in objective response rate or progression-free survival based on platinum resistant or heavily pretreated status. Furthermore, an initial dose of lenvatinib at 10 mg or less was non-inferior compared with higher starting doses. These findings should not be interpreted as demonstrating inherent non-inferiority of lower starting doses given this is a small retrospective review and confidence intervals of the response rate were wide. Despite the heavily pretreated, platinum-resistant, high-grade population, these findings demonstrate there is clinical activity starting with a reduced dose of lenvatinib. This study represents, to our knowledge, the first report at this dose in this setting.

Additional findings included no statistically significant benefit in dMMR versus pMMR, we believe this occurred due to the low number of dMMR patients within our retrospective review.

### 4.2 Results in the context of published literature

The phase 3 KEYNOTE-3 775/ Study 309 was a pivotal study that confirmed the survival benefit of lenvatinib plus pembrolizumab in patients with advanced endometrial cancer and mismatch repair-proficient tumors who had received at least one prior line of platinum-based therapy. The Phase 3 trial initially reported an objective response rate of 31.9%, later updated to 33.8%. With most endometrial tumors being MMR proficient, that study changed the standard of care for advanced and recurrent endometrial cancer in this subtype. However, the population included only had one prior line of platinum-based therapy, without clarification on platinum resistance status following the first line chemotherapy. Additionally, the cohort included a healthy patient population (ECOG 0–1). We believe our real-world data helps illustrate the efficacy and decreased toxicity with lower doses of lenvatinib in heavily pretreated high-grade, platinum-resistant, P53 mutated uterine cancers (Makker et al., 2023, Makker et al., 2022, Ozawa et al., 2024).

Serous, P53 mutated endometrial cancer accounted for 67.8% of our cohort, compared to 28.6% in the KEYNOTE-775 trial. Among our serous endometrial cancer patients, 4 achieved a complete response and 13 had a partial response, yielding an objective response rate of 42.5% (Table 2). Additionally, our cohort included 11 patients with carcinosarcoma (19%) with one achieving a complete response, and one a partial response (objective rate response 18%, clinical benefit rate 36%). These findings support the potential role of immunotherapy with lenvatinib in hard-to-treat endometrial cancer subtypes with a poor prognosis.

While KEYNOTE-775 (Study 309) demonstrated survival benefits, lenvatinib at the recommended 20 mg dose often causes significant toxicity, necessitating dose reductions, treatment delays, or hospital admissions. The dose of 20 mg was determined based on a phase 1b trial of Lenvatinib and pembrolizumab in patients with selected solid tumors. The study compared starting doses of lenvatinib at 24 mg and 20 mg. From 13 patients, the 20 mg starting dose did not have any dose-limiting toxicities and was determined to be the maximum tolerated dose (Taylor et al., 2016). However, in KEYNOTE-775/Study 309, 66.5% of patients required a dose reduction of lenvatinib, 69.2% of patients had an interruption of treatment, and 33.0% of patients discontinued therapy (Makker et al., 2022).

Prior retrospective data have demonstrated comparable efficacy with reduced starting doses of lenvatinib at 14 mg, while showing improved tolerability compared with the recommended dose (How et al., 2021). However, these studies did not stratify patients by platinum resistance or p53 mutation status, limiting their applicability to biologically high-risk populations. Our study builds on this literature by evaluating reduced-dose lenvatinib, at lower doses than the reported 14 mg, in a cohort enriched for platinum-resistant, p53-mutated disease and hard to treat uterine carcinosarcomas.

In the prior retrospective data, it is important to note that How et al. did not define or stratify patients by platinum sensitivity or p53 mutation status. As a result, the proportions of platinum-sensitive versus platinum-resistant patients, as well as p53 status, remain unknown. Any imbalance between treatment arms (14 mg vs. 20 mg) could have potentially confounded their findings. In contrast, our cohort was enriched for platinum-resistant, p53-mutated, carcinosarcoma and high-grade cases, and outcomes were reported with stratification by these characteristics. Moreover, while How et al. observed no statistically significant difference between the 14 mg and 20 mg cohorts, they did not perform a non-inferiority analysis. In our retrospective study, non-inferiority was assessed, demonstrating that reduced starting doses (≤10 mg) achieved comparable efficacy with improved tolerability.

At our institution, patients are initiated on doses lower than the traditional 14 or 20 mg due to toxicity concerns, especially in heavily pretreated patients with higher ECOG scores. Our data demonstrated that the longest-tolerated dose was 10 mg with a median duration of 74 days. We subsequently stratified outcomes by starting doses of ≤ 10 mg and > 10 mg. Like the How et. al. study, we found no significant difference in objective response rate or progression-free survival, indicating that patients on the lower dose were still achieving clinical benefit from the combination therapy. We further explored this question using an exploratory non-inferiority framework, which suggested no evidence of inferior efficacy for starting doses of ≤ 10 mg compared with higher doses. Given the retrospective design and limited sample size of this study, these findings should be interpreted as hypothesis-generating and require validation in larger prospective studies, as well as randomized controlled trials that compare ≤ 10 mg lenvatinib to higher doses. The reduced dose group also had a smaller proportion of dose discontinuations, dose reductions and dose interruptions, along with lower rates of hypertension, fatigue, nausea and mucositis, although not statistically significant. However, larger studies are necessary to determine the non-inferiority of dose reduction compared to the current standard recommended dose of lenvatinib.

An interesting and novel finding in this study was the effect of p53 status on progression-free survival. On subgroup analysis, p53 mutation was associated with improved progression free survival with lenvatinib and pembrolizumab compared with non p53 mutated tumors, corresponding to a 60.7% reduction in the risk of progression or death (HR 0.393, 95% CI 0.167 to 0.926). This association persisted on multivariable analysis. Makker et. al. evaluated potential biomarkers for lenvatinib and pembrolizumab from the data obtained in phase Ib/II KEYNOTE-146/ Study 111. Their study found no significant difference in objective response rate based on p53 mutation status from the general population and based on microsatellite stable status (Makker et al., 2024). This discrepancy may be explained by our use of progression-free survival as a measure. Inherently progression-free survival includes patients with stable disease, potentially leading to longer disease-free progression, but not showing a benefit in ORR. Conversely, Makker et al. focused on ORR, which only captures partial and complete responses, (stable disease is unaccounted for in ORR). However, additional studies would be necessary to further review p53 mutational status as a potential biomarker to predict clinical response to pembrolizumab and lenvatinib. Despite the TP53 mutations being among the most identified in cancers, there is currently no standard targeted therapy for this mutation. Prior studies have indicated some promise with vascular endothelial growth factor (VEGF) inhibitors, as TP53 notably increases vascular endothelial growth factor (VEGF) expression in tumors (Wheler et al., 2016).

### 4.3 Strengths and weaknesses

A strength of our study was the representation of patients with platinum-resistant, P53 mutant disease with a large number of patients afflicted with carcinosarcoma. Another strength of our study was representing real world consideration of toxicity of lenvatinib by evaluating lower starting doses, especially in a heavily pre-treated patients with higher ECOG scores.

As a retrospective study, there is potential for missing data due to lack of systematic documentation of toxicity, as it was based on each patient’s subjective reports of their symptoms, vital signs, and laboratory values. The number of variables included in exploratory multivariable analyses relative to the sample size may increase the risk of model overfitting, and these findings should therefore be interpreted cautiously. Moreover, lenvatinib dosing was at the discretion of the treating gynecologic oncologist, introducing an opportunity for selection bias. The population was limited in size and not powered to detect significant differences in objective response, progression-free survival, hospitalization rates, dose reductions, and side effects. Additionally, lenvatinib starting dose was not randomly assigned, and clinician-driven treatment selection based on anticipated tolerability may have introduced residual confounding that could influence comparisons between dose groups.

### 4.4 Implications for practice and future research

This study is the first to show that lower doses at 10 mg or less were better tolerated and had non-inferior clinical efficacy when compared to the higher recommended doses of lenvatinib. These data complement prior evidence for the efficacy of pembrolizumab and lenvatinib in mismatch repair proficient advanced and recurrent high grade endometrial cancer, including uterine carcinosarcoma. This study also reinforces existing evidence that initiating lenvatinib at a lower dose may improve tolerability without compromising efficacy, particularly in heavily pretreated patients with higher baseline ECOG performance status. Our study demonstrates p53 as a potential biomarker to predict response to this combination therapy. Larger studies are required to determine the optimal starting dose of lenvatinib to improve tolerability while maintaining efficacy and validate p53 as a predictive biomarker. This data could guide clinicians in determining next steps when comparing therapeutic options for recurrent endometrial cancer.

## CRediT authorship contribution statement

**Alexa Dzienny:** Writing – original draft, Visualization, Validation, Methodology, Investigation, Formal analysis, Data curation. **Yasmin Abozenah:** Writing – review & editing, Visualization, Project administration, Methodology, Investigation, Formal analysis. **James Griffith:** Formal analysis. **Sidra Sohail:** Formal analysis. **Yifang Eva Pan:** Writing – review & editing, Investigation, Formal analysis, Data curation. **Christina Vlamis:** Writing – review & editing, Data curation. **Katelyn Yamartino:** Writing – original draft. **Jessica Leclair:** Writing – review & editing, Formal analysis, Data curation. **Gary Israel:** Writing – review & editing. **Michelle Greenman:** Writing – review & editing. **Blair McNamara:** Writing – review & editing. **Masoud Azodi:** Writing – review & editing. **Elena S. Ratner:** Writing – review & editing. **Alessandro Santin:** Writing – review & editing. **Peter Dottino:** Writing – review & editing. **Katyayani Papatla:** Writing – review & editing. **Aparna Kailasam:** Writing – review & editing. **Pei Hui:** Writing – review & editing. **Natalia Buza:** Writing – review & editing. **Gary Altwerger:** Writing – review & editing, Validation, Formal analysis, Data curation, Conceptualization.

## Declaration of competing interest

The authors declare that they have no known competing financial interests or personal relationships that could have appeared to influence the work reported in this paper.
