# Supplementary material for: Real World Outcomes of Pembrolizumab and Reduced-Dose Lenvatinib in Recurrent Endometrial Cancer by Platinum and p53 Status
Source: Gynecol Oncol Rep. 2026 Apr 15;65:102084. doi: 10.1016/j.gore.2026.102084 (PMC13139971; doi:10.1016/j.gore.2026.102084)
Supplement: Supplementary Data 1 [file mmc1.pptx]

## Slide 1
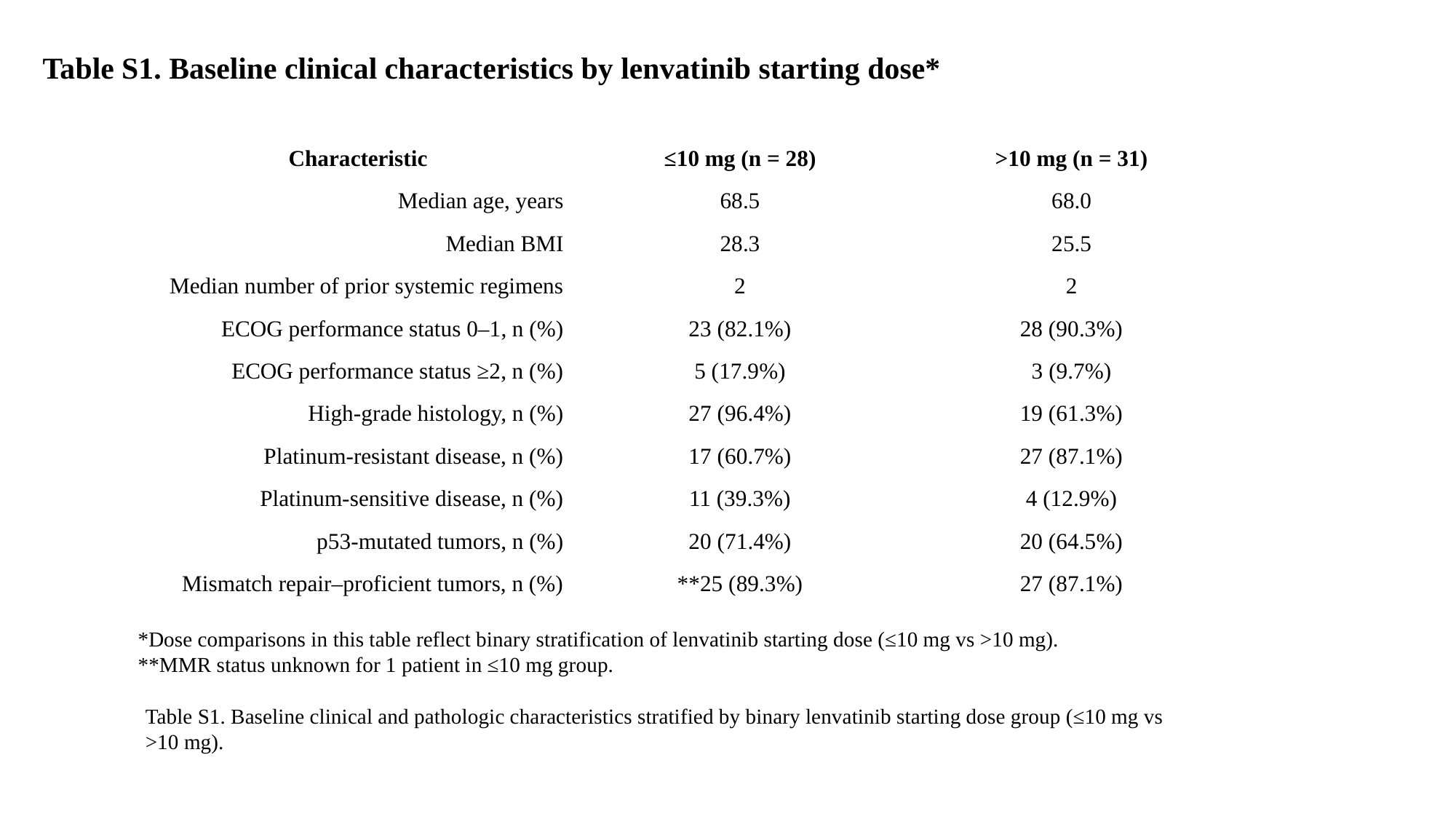

Table S1. Baseline clinical characteristics by lenvatinib starting dose*
| Characteristic | ≤10 mg (n = 28) | >10 mg (n = 31) |
| --- | --- | --- |
| Median age, years | 68.5 | 68.0 |
| Median BMI | 28.3 | 25.5 |
| Median number of prior systemic regimens | 2 | 2 |
| ECOG performance status 0–1, n (%) | 23 (82.1%) | 28 (90.3%) |
| ECOG performance status ≥2, n (%) | 5 (17.9%) | 3 (9.7%) |
| High-grade histology, n (%) | 27 (96.4%) | 19 (61.3%) |
| Platinum-resistant disease, n (%) | 17 (60.7%) | 27 (87.1%) |
| Platinum-sensitive disease, n (%) | 11 (39.3%) | 4 (12.9%) |
| p53-mutated tumors, n (%) | 20 (71.4%) | 20 (64.5%) |
| Mismatch repair–proficient tumors, n (%) | \*\*25 (89.3%) | 27 (87.1%) |
*Dose comparisons in this table reflect binary stratification of lenvatinib starting dose (≤10 mg vs >10 mg).
**MMR status unknown for 1 patient in ≤10 mg group.
Table S1. Baseline clinical and pathologic characteristics stratified by binary lenvatinib starting dose group (≤10 mg vs >10 mg).

## Slide 2
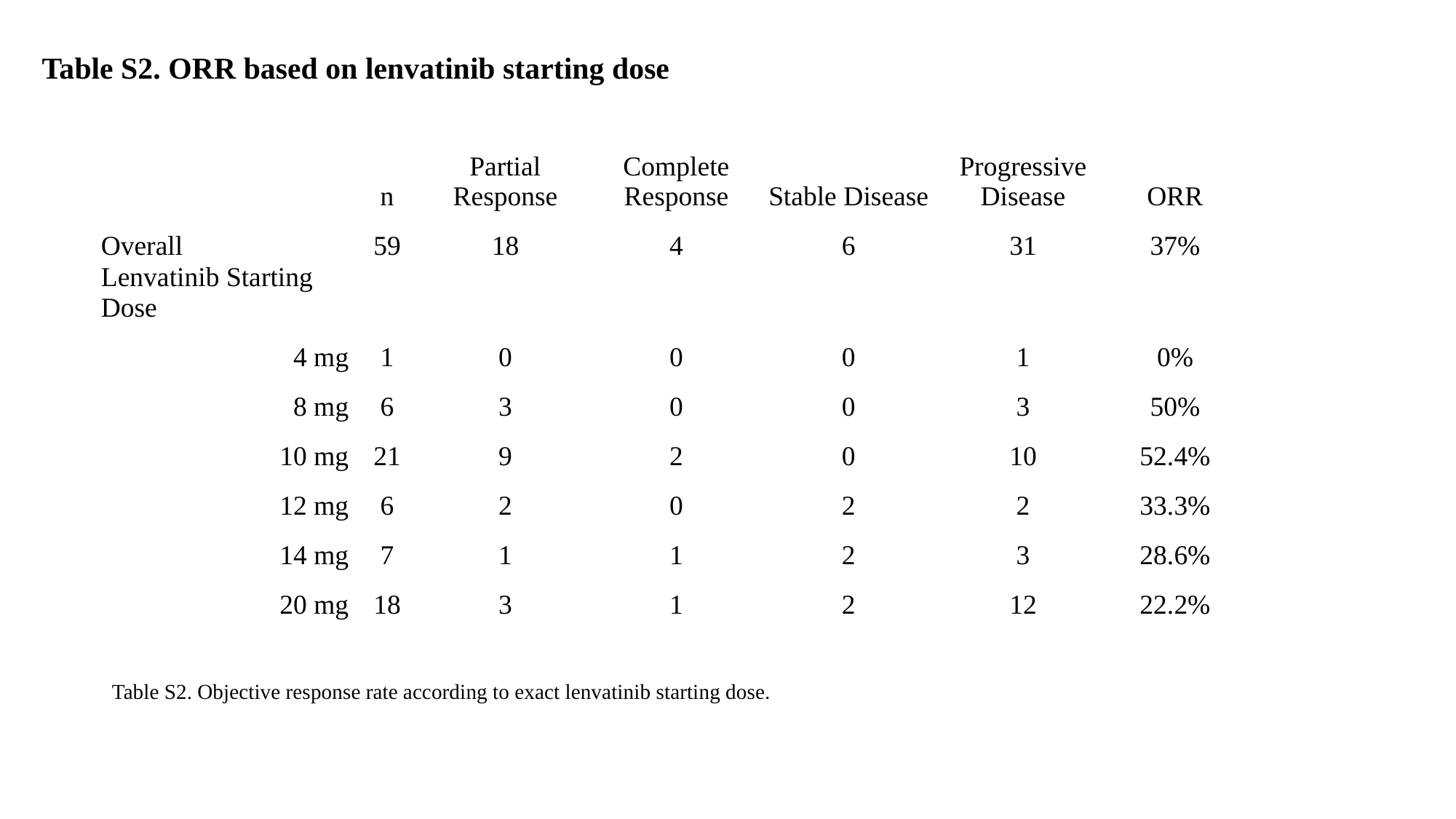

Table S2. ORR based on lenvatinib starting dose
| | n | Partial Response | Complete Response | Stable Disease | Progressive Disease | ORR | |
| --- | --- | --- | --- | --- | --- | --- | --- |
| Overall | 59 | 18 | 4 | 6 | 31 | 37% | |
| Lenvatinib Starting Dose | | | | | | | |
| 4 mg | 1 | 0 | 0 | 0 | 1 | 0% | |
| 8 mg | 6 | 3 | 0 | 0 | 3 | 50% | |
| 10 mg | 21 | 9 | 2 | 0 | 10 | 52.4% | |
| 12 mg | 6 | 2 | 0 | 2 | 2 | 33.3% | |
| 14 mg | 7 | 1 | 1 | 2 | 3 | 28.6% | |
| 20 mg | 18 | 3 | 1 | 2 | 12 | 22.2% | |
Table S2. Objective response rate according to exact lenvatinib starting dose.

## Slide 3
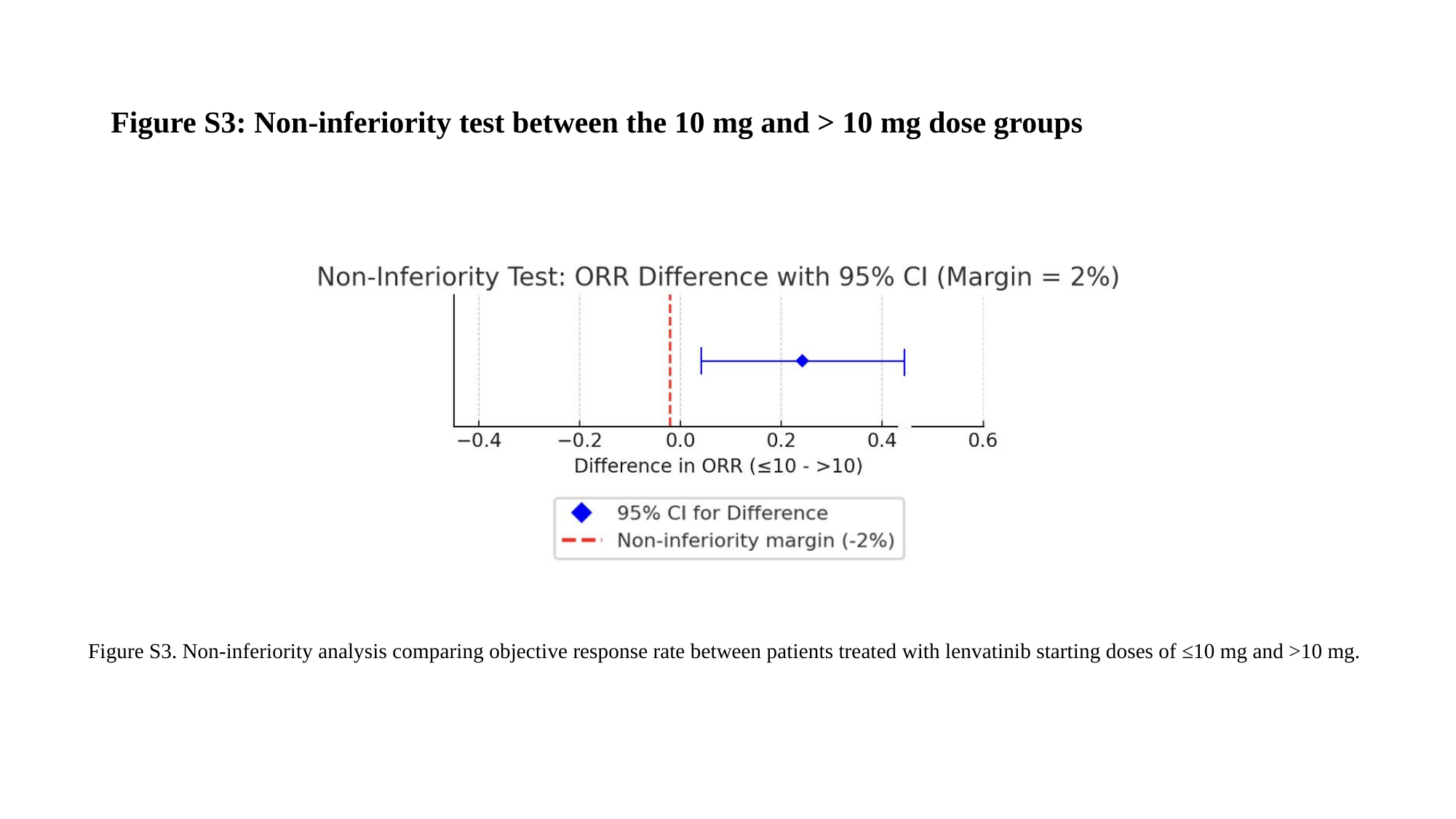

Figure S3. Non-inferiority analysis comparing objective response rate between patients treated with lenvatinib starting doses of ≤10 mg and >10 mg.

## Slide 4
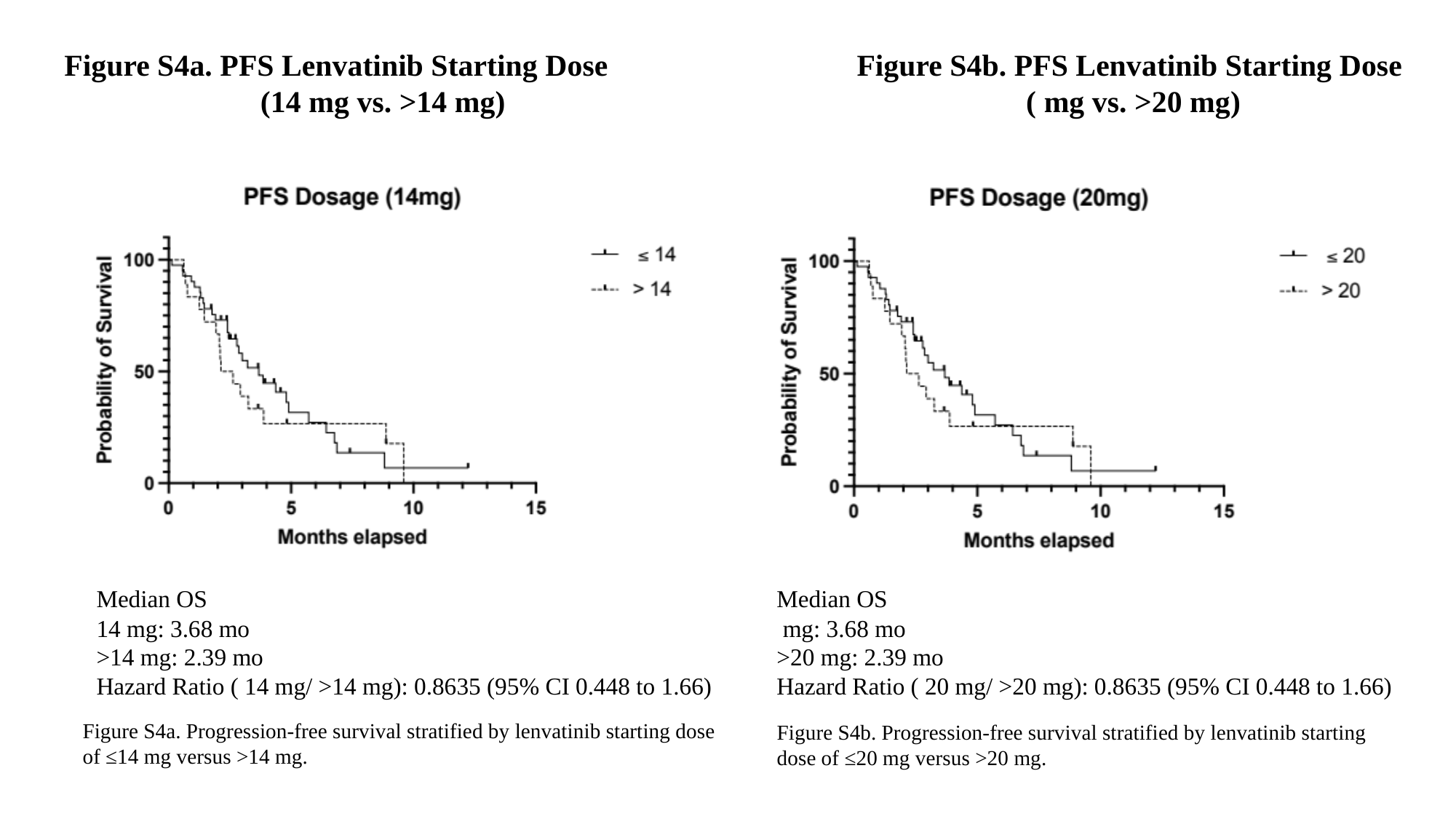

Figure S4a. Progression-free survival stratified by lenvatinib starting dose of ≤14 mg versus >14 mg.
Figure S4b. Progression-free survival stratified by lenvatinib starting dose of ≤20 mg versus >20 mg.

## Slide 5
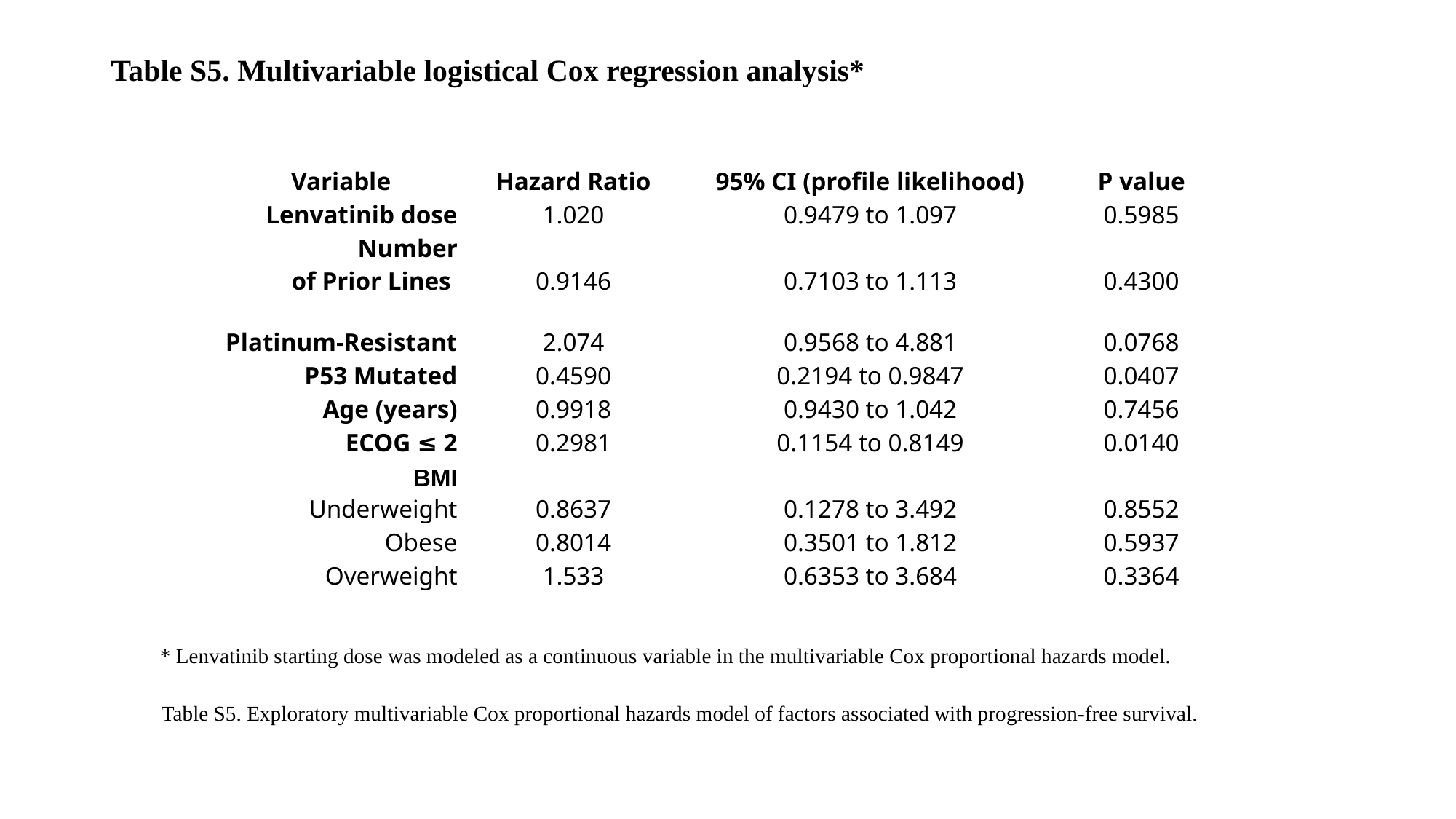

# Table S5. Multivariable logistical Cox regression analysis*
| Variable | Hazard Ratio | 95% CI (profile likelihood) | P value |
| --- | --- | --- | --- |
| Lenvatinib dose | 1.020 | 0.9479 to 1.097 | 0.5985 |
| Number of Prior Lines | 0.9146 | 0.7103 to 1.113 | 0.4300 |
| Platinum-Resistant | 2.074 | 0.9568 to 4.881 | 0.0768 |
| P53 Mutated | 0.4590 | 0.2194 to 0.9847 | 0.0407 |
| Age (years) | 0.9918 | 0.9430 to 1.042 | 0.7456 |
| ECOG ≤ 2 | 0.2981 | 0.1154 to 0.8149 | 0.0140 |
| BMI | | | |
| Underweight | 0.8637 | 0.1278 to 3.492 | 0.8552 |
| Obese | 0.8014 | 0.3501 to 1.812 | 0.5937 |
| Overweight | 1.533 | 0.6353 to 3.684 | 0.3364 |
* Lenvatinib starting dose was modeled as a continuous variable in the multivariable Cox proportional hazards model.
Table S5. Exploratory multivariable Cox proportional hazards model of factors associated with progression-free survival.

## Slide 6
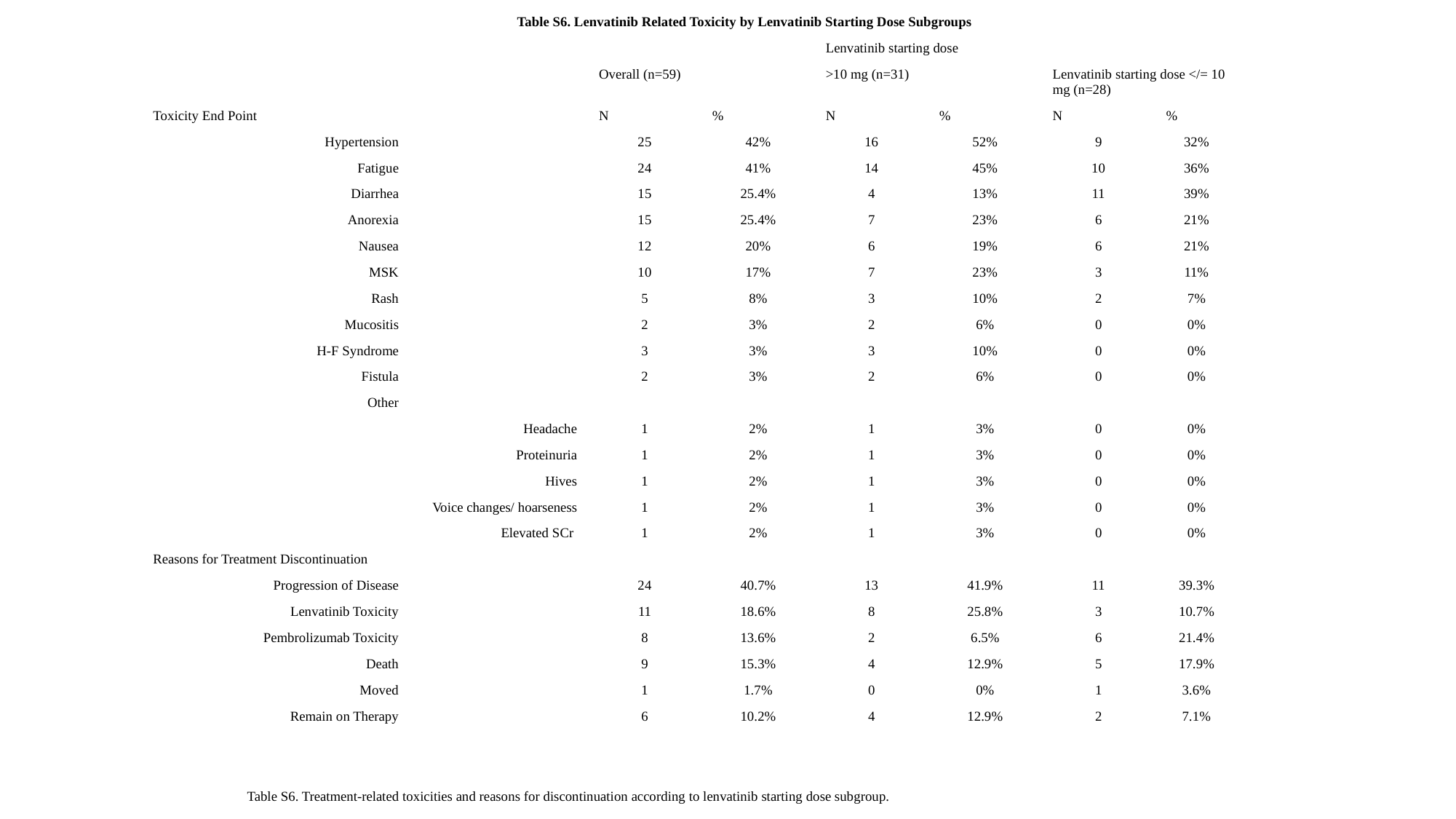

| Table S6. Lenvatinib Related Toxicity by Lenvatinib Starting Dose Subgroups | | | | | | | | |
| --- | --- | --- | --- | --- | --- | --- | --- | --- |
| | | | | Lenvatinib starting dose | | | | |
| | | Overall (n=59) | | >10 mg (n=31) | | Lenvatinib starting dose </= 10 mg (n=28) | | |
| Toxicity End Point | | N | % | N | % | N | % | |
| Hypertension | | 25 | 42% | 16 | 52% | 9 | 32% | |
| Fatigue | | 24 | 41% | 14 | 45% | 10 | 36% | |
| Diarrhea | | 15 | 25.4% | 4 | 13% | 11 | 39% | |
| Anorexia | | 15 | 25.4% | 7 | 23% | 6 | 21% | |
| Nausea | | 12 | 20% | 6 | 19% | 6 | 21% | |
| MSK | | 10 | 17% | 7 | 23% | 3 | 11% | |
| Rash | | 5 | 8% | 3 | 10% | 2 | 7% | |
| Mucositis | | 2 | 3% | 2 | 6% | 0 | 0% | |
| H-F Syndrome | | 3 | 3% | 3 | 10% | 0 | 0% | |
| Fistula | | 2 | 3% | 2 | 6% | 0 | 0% | |
| Other | | | | | | | | |
| | Headache | 1 | 2% | 1 | 3% | 0 | 0% | |
| | Proteinuria | 1 | 2% | 1 | 3% | 0 | 0% | |
| | Hives | 1 | 2% | 1 | 3% | 0 | 0% | |
| | Voice changes/ hoarseness | 1 | 2% | 1 | 3% | 0 | 0% | |
| | Elevated SCr | 1 | 2% | 1 | 3% | 0 | 0% | |
| Reasons for Treatment Discontinuation | | | | | | | | |
| Progression of Disease | | 24 | 40.7% | 13 | 41.9% | 11 | 39.3% | |
| Lenvatinib Toxicity | | 11 | 18.6% | 8 | 25.8% | 3 | 10.7% | |
| Pembrolizumab Toxicity | | 8 | 13.6% | 2 | 6.5% | 6 | 21.4% | |
| Death | | 9 | 15.3% | 4 | 12.9% | 5 | 17.9% | |
| Moved | | 1 | 1.7% | 0 | 0% | 1 | 3.6% | |
| Remain on Therapy | | 6 | 10.2% | 4 | 12.9% | 2 | 7.1% | |
Table S6. Treatment-related toxicities and reasons for discontinuation according to lenvatinib starting dose subgroup.
